# Estimated Number Needed to Treat to Avoid a First Hospitalization by Maintaining Instead of Reducing Renin-Angiotensin-Aldosterone System Inhibitor (RAASi) Therapy after Hyperkalemia

**DOI:** 10.34067/KID.0000000000000561

**Published:** 2024-08-21

**Authors:** Maria K. Svensson, Michael Fischereder, Paul R. Kalra, Ignacio José Sánchez Lázaro, Eva Lesén, Stefan Franzén, Alaster Allum, Thomas Cars, Nils Kossack, Philipp Breitbart, David Arroyo

**Affiliations:** 1 Department of Medical Sciences, Renal Medicine, Uppsala University, Uppsala, Sweden; 2 Uppsala Clinical Research Centre, Uppsala University, Uppsala, Sweden; 3 Division of Nephrology, Medizinische Klinik und Poliklinik IV, LMU Klinikum, LMU Munich, Munich, Germany; 4 Department of Cardiology, Portsmouth Hospitals University NHS Trust, Portsmouth, United Kingdom; 5 Cardiology Department, Hospital Universitari i Politècnic La Fe, Valencia, Spain; 6 CVRM Evidence Strategy, BioPharmaceuticals Medical, AstraZeneca, Gothenburg, Sweden; 7 BPM Evidence Statistics, Medical Evidence, BioPharmaceuticals Medical, AstraZeneca, Gothenburg, Sweden; 8 BioPharmaceuticals Medical CVRM, AstraZeneca, Cambridge, United Kingdom; 9 Sence Research AB, Uppsala, Sweden; 10 WIG2 Scientific Institute for Health Economics and Health System Research, Leipzig, Germany; 11 Department of Cardiology and Angiology, Medical Center, Faculty of Medicine, University of Freiburg, Bad Krozingen, Germany; 12 Nephrology Department, Hospital General Universitario Gregorio Marañón, Madrid, Spain

**Keywords:** ACE inhibitors, CKD, clinical epidemiology, electrolytes, epidemiology and outcomes, heart failure, hospitalization, renin angiotensin system

## Abstract

**Key Points:**

- Renin-angiotensin-aldosterone system inhibitor (RAASi) therapy is frequently downtitrated or discontinued after a hyperkalemia episode.
- Reducing RAASi therapy after a hyperkalemia episode is associated with increased risk of hospitalization compared with maintaining RAASi.
- Our data suggest that a hospitalization within 6 months could be avoided if 25 patients maintained instead of reduced their RAASi therapy.

**Background:**

Renin-angiotensin-aldosterone system inhibitor (RAASi) therapy provides cardiorenal protection but is often downtitrated or discontinued after a hyperkalemia episode. This observational study describes the extent of hyperkalemia-related RAASi reduction in patients with CKD and/or heart failure (HF) and estimates the number needed to treat (NNT) to avoid a first hospitalization if RAASi had been maintained at the prior dose.

**Methods:**

Health care registers and claims data from Germany, Spain, Sweden, and the United Kingdom were used to identify nondialysis patients with CKD and/or HF who had a hyperkalemia episode while on RAASi. Patients whose RAASi therapy was reduced (downtitrated/discontinued) after the hyperkalemia episode were propensity score matched to those with maintained RAASi, and their risks of a hospitalization within 6 months were estimated using the Kaplan–Meier method. On the basis of the absolute difference in this 6-month risk, the NNT framework was applied to estimate the number of patients who needed to have maintained instead of reduced their RAASi to avoid a first hospitalization during this period.

**Results:**

Overall, 40,059 patients from Germany, Spain, Sweden, and the United Kingdom were included. Presence of CKD at baseline was similar across countries (72%–92%), while HF was less common in Spain (18%) versus other countries (32%–71%). After the hyperkalemia episode, RAASi was reduced in 25%–57% of patients. After propensity score matching, the 6-month risk of hospitalization was consistently higher in those with reduced versus maintained RAASi; the absolute risk difference ranged from 2.7% to 7.3%. Applying the NNT framework, these data suggest that a first hospitalization within 6 months could potentially have been avoided if 25 patients had maintained instead of reduced their RAASi.

**Conclusions:**

Our findings suggest a potential for avoiding a first hospitalization, even within a short time frame, by increasing adherence to guidelines to maintain instead of reduce RAASi after a hyperkalemia episode.

## Visual Abstract

**Figure:**
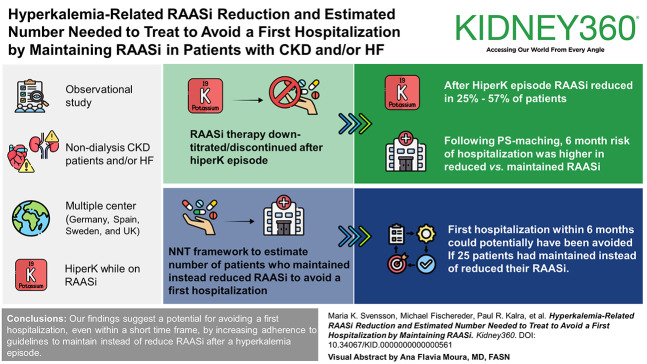

## Introduction

Renin-angiotensin-aldosterone system inhibitors (RAASis) provide cardiorenal protection by reducing the risk of cardiovascular mortality and morbidity, as well as progression of renal dysfunction toward kidney failure, in patients with heart failure (HF) or CKD.^1,2^ International evidence-based guidelines recommend RAASi medication at the maximal tolerated dose to optimize treatment benefits.^3–5^ However, RAASi medication also increases the risk of hyperkalemia (often defined as serum potassium [K^+^] >5.0 mmol/L).^3–7^ The risk of developing hyperkalemia is twice as high among patients receiving RAASi therapy compared with those not receiving RAASi therapy.^8^ If left untreated, hyperkalemia can lead to adverse outcomes, including hospitalization and, in severe cases, cardiac arrhythmia, cardiac arrest, and sudden death.^9–11^ In clinical practice, RAASi therapy is often downtitrated or discontinued in patients with hyperkalemia.^12–15^ Hence, hyperkalemia or the risk of hyperkalemia may be a significant barrier to use of evidence-based and guideline-directed medical treatment.

Until recently, longer term management of hyperkalemia typically involved reducing or discontinuing RAASi therapy, restricting dietary K^+^, or use of diuretic therapy. Acute management also included traditional K^+^ binders (*e.g*., sodium or calcium polystyrene sulfonate), although their use is hampered by adverse gastrointestinal side effects, lack of palatability, limited evidence on longer term efficacy, and poor adherence to treatment.^12–14,16–20^ More recently, the newer K^+^ binders patiromer and sodium zirconium cyclosilicate have become available. International guidelines and consensus from practicing clinicians now suggest targeted treatment with these K^+^ binders to facilitate the maintenance of RAASi therapy and that RAASi therapy should be reduced or discontinued only if such mitigation strategies have not been sufficient.^3–5,21,22^ Previous studies have described the increased risk of adverse cardiorenal outcomes associated with not attaining maximum target dosing or with discontinuation of RAASi therapy.^6,7,16,23^ However, studies evaluating the potential benefits of maintaining RAASi therapy at a prior dose, rather than reducing therapy after a hyperkalemia episode, are scarcer.^15,24^ Such studies are important to reinforce the guideline recommendations to maintain RAASi therapy in patients after a hyperkalemia episode, support practice change, and, thereby, optimize treatment benefits.

The aim of this observational cohort study was to assess the extent of hyperkalemia-related RAASi reduction and subsequent reinitiation among European patients with cardiorenal disease and to assess the risk of hospitalizations in those with reduced versus maintained RAASi therapy. To give clinicians a sense of the magnitude of impact, this study also estimated the number needed to treat (NNT) to avoid a first hospitalization if RAASi therapy had instead been maintained at the prior dose.

## Methods

### Data Sources

This study is part of ZORA, an observational study program investigating the management and consequences of hyperkalemia in cardiorenal patients in routine clinical practice.^15,24,25^ This study used retrospectively collected data from health care claims, registries, and medical records from four European countries: Germany, Spain, Sweden, and the United Kingdom. The data sources are detailed in the Supplemental Material. In brief, the German WIG2 benchmark database contains routinely collected data from a representative sample of patients insured by the German statutory health insurance^26^; the Spanish BIG-PAC database captures electronic medical records data from seven Spanish regions^27^; in Sweden, electronic medical records data from two regions were linked to national registries capturing data on prescriptions, secondary care, and death registrations; the UK Clinical Practice Research Datalink Aurum database with primary care data was linked to data sources capturing secondary care and death registrations.^28^

### Study Population

The study population included adult nondialysis patients with CKD and/or HF who had a hyperkalemia episode while on RAASi therapy. The overall study period spanned from January 2018 to December 2022; country-specific study periods are detailed in the Supplemental Material. Eligible hyperkalemia episodes were recorded in inpatient or specialist outpatient care and defined by a recorded International Classification of Diseases 10th Revision (ICD-10) code E87.5 (in all countries) or as a recorded K^+^ value >5.0 mmol/L in data sources capturing such laboratory data (Spain and Sweden); Swedish data also captured hyperkalemia episodes in nonspecialist outpatient care. The index date was defined as the date of the recorded hyperkalemia episode if recorded in the outpatient setting or the date of discharge if recorded in the inpatient setting. For patients with multiple eligible index dates, the index episode was defined as a randomly selected hyperkalemia episode that met the eligibility criteria, with hyperkalemia episodes recorded using a diagnosis code taking precedence over a recorded laboratory measurement. Hyperkalemia episodes preceded by <12 months of available lookback data before the index episode (for assessment of baseline characteristics) were not eligible for inclusion.

Patients were required to have a recorded ICD-10 diagnosis code for HF and/or CKD or eGFR <60 ml/min per 1.73 m^2^ (Sweden and the United Kingdom only), but no dialysis, within 12 months before the index date (or any time before the index date in Sweden). Patients were also required to have an issued or filled prescription for at least one RAASi medication within 120 days before the index date. The RAASi medication classes included angiotensin-converting enzyme inhibitors (ACEis), angiotensin-receptor blockers (ARBs), mineralocorticoid receptor antagonists (MRAs), and angiotensin receptor neprilysin inhibitors.

Patients were defined as having reduced (downtitrated or discontinued) versus maintained (including uptitration) their RAASi therapy after the index hyperkalemia episode on the basis of their most recent RAASi prescriptions within the 120 days before versus the 120 days after the index date. Reduced RAASi therapy was defined as discontinuation (no prescription for any RAASi class) or downtitration (use of fewer RAASi classes or when the dose of at least one preindex RAASi class was reduced by ≥25% after the index date). Maintained RAASi therapy was defined as having postindex prescription(s) for at least the same number of RAASi classes as preindex. To avoid misclassification bias, patients with <120 days of available follow-up time after the index date, due to censoring or death, were therefore excluded.

### Extent of RAASi Reduction and Subsequent Reinitiation

The proportion and baseline characteristics of patients with reduced versus maintained RAASi therapy were described. RAASi discontinuation was further described as discontinuation of all RAASi therapy and discontinuation of each RAASi class. Reinitiation of any RAASi therapy and of each RAASi class was described within 1 year of the hyperkalemia episode in those with at least 1 year of available follow-up time.

### Risk of Hospitalizations in Patients with Reduced versus Maintained RAASi

Within each country, propensity score (PS) matching was applied to balance the cohort of patients with reduced RAASi to the cohort of patients with maintained RAASi on potential baseline confounders. The covariates evaluated for inclusion in the PS matching covered baseline demographics, comorbidities, comedications (including RAASi type and dose), health care resource use, K^+^ value at the index date (Spain and Sweden), history of K^+^ binder use, and eGFR (except in Germany). The full list of covariates evaluated for inclusion in the PS matching is specified in the Supplemental Material.

Logistic regression modeling was used for PS estimation, with a caliper of 0.10 SD of the logit PS. A PS matching ratio of 1:1 or 1:2 was applied as applicable in each dataset. Covariates with a standardized mean difference (SMD) <10% after matching were considered balanced.

In the PS-matched cohorts of patients with reduced versus maintained RAASi therapy, the 6-month risk and 95% confidence interval (CI) of an all-cause hospitalization was assessed using the Kaplan–Meier method. In addition, the total number of hospitalizations during 6 months of follow-up was described as the all-events rate per person-year of follow-up, along with the Poisson exact 95% CI. Patients were censored at the end of follow-up or death.

The absolute risk differences and 95% CIs of the 6-month risk of a hospitalization were meta-analyzed across countries using a random-effects model. The NNT framework^29,30^ was then applied to estimate the number of patients who would need to have maintained, instead of reduced, their prior RAASi therapy to avoid a first hospitalization within 6 months.

## Results

### Study Population

In total, 40,059 patients from Germany, Spain, Sweden, and the United Kingdom were included. The patient attrition flow charts are shown in Supplemental Figure 1, and baseline characteristics are presented in Table 1. Across countries, the mean age was 75.7−78.6 years, and 39.4%–56.1% were male. Presence of CKD at baseline was similar across countries (72.0%–91.6%), while HF was more common in Germany (71.3%), Sweden (55.3%), and the United Kingdom (32.2%) than in Spain (17.9%). ACEi and ARB were generally the most commonly prescribed RAASi classes at baseline, with ARB particularly common in Spain. In Germany, Sweden, and the United Kingdom, approximately one third of patients used MRA (27.5%–34.9%), although this was less common in Spain (15.8%).

**Table 1:** Baseline characteristics of patients with CKD and/or HF who had a hyperkalemia episode

Characteristic	Germany (*n*=11,677)	Spain (*n*=4392)	Sweden (*n*=17,404)	United Kingdom (*n*=6586)
Age, yr, mean (SD)	75.8 (10.7)	78.6 (9.6)	75.7 (11.5)	76.3 (11.7)
Male, No. (%)	4601 (39.4)	2231 (50.8)	7328 (42.1)	3698 (56.1)
**Hyperkalemia severity at index, No. (%)**				
Mild^a^	n/a	2214 (50.4)	11,527 (67.0)	n/a
Moderate^a^	n/a	1642 (37.4)	3426 (19.9)	n/a
Severe^a^	n/a	536 (12.2)	2241 (13.0)	n/a
Missing	11,677 (100.0)	0 (0.0)	210 (1.2)	6586 (100.0)
**CKD, No. (%)**	8402 (72.0)	3742 (85.2)	14,407 (82.8)	6035 (91.6)
Stage 3	4334 (37.1)	2994 (68.2)	9938 (57.1)	3340 (50.7)
Stage 4	1957 (16.8)	558 (12.7)	3427 (19.7)	1965 (29.8)
Stage 5	405 (3.5)	84 (1.9)	1042 (6.0)	518 (7.9)
Unknown stage	1706 (14.6)	106 (2.4)	0 (0.0)	212 (3.2)
HF, No. (%)	8325 (71.3)	786 (17.9)	9622 (55.3)	2121 (32.2)
Diabetes, No. (%)	7133 (61.1)	1913 (43.6)	8089 (46.5)	3662 (55.6)
**RAASi, No. (%)**				
ACEi	5786 (49.6)	1896 (43.2)	8602 (49.4)	4190 (63.6)
ARB	4460 (38.2)	2422 (55.1)	7647 (43.9)	2026 (30.8)
ARNi	838 (7.2)	210 (4.8)	494 (2.8)	97 (1.5)
MRA	4076 (34.9)	695 (15.8)	5267 (30.3)	1811 (27.5)

ACEi, angiotensin-converting enzyme inhibitor; ARB, angiotensin receptor blocker; ARNi, angiotensin receptor neprilysin inhibitor; HF, heart failure; MRA, mineralocorticoid receptor antagonist; n/a, not applicable; RAASi, renin-angiotensin-aldosterone system inhibitor.

a Percentages were calculated using patients with nonmissing data as the denominator; hyperkalemia severity was defined as follows: mild, >5.0 to <5.5; moderate, 5.5 to <6.0; and severe ≥6.0 mmol/L.

### Extent of RAASi Reduction

After the hyperkalemia episode, RAASi therapy was reduced (downtitrated or discontinued) in 44.4% (Germany), 24.8% (Spain), 34.7% (Sweden), and 57.1% (the United Kingdom) of patients. Baseline characteristics of patients with reduced versus maintained RAASi therapy are presented in Supplemental Table 1 (before PS matching). In Germany, Sweden, and the United Kingdom, baseline MRA use was more common in those with reduced versus maintained RAASi therapy. In Spain, MRA use was less frequent overall (as noted above) and similar between the two groups, whereas ARB use was more common overall and especially among those with reduced versus maintained RAASi therapy; ACEi use was less common in those with reduced RAASi.

### RAASi Discontinuation and Reinitiation

**Figure 1:**
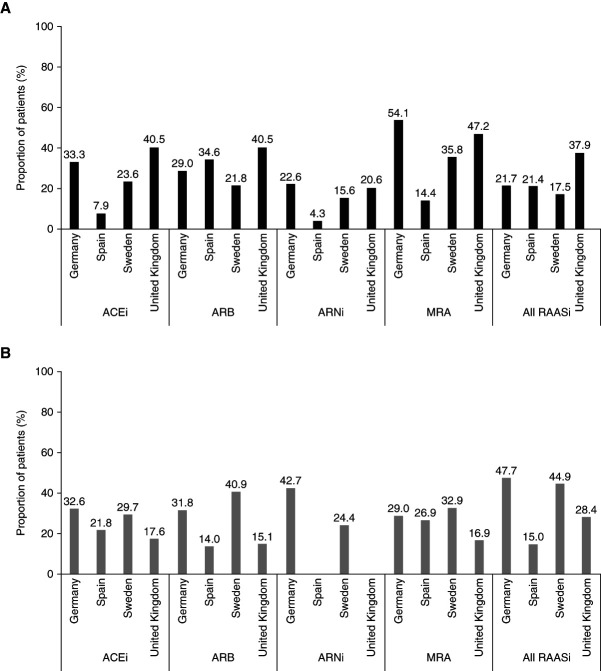
**RAASi discontinuation and reinitiation.** RAASi (A) discontinuation after an index hyperkalemia episode and (B) reinitiation within 1 year after an index hyperkalemia episode. The percentage of patients who reinitiated within 1 year was calculated using the number of patients with at least 365 days of available follow-up as the denominator. Patient numbers are detailed in Supplemental Table 2. ACEi, angiotensin-converting enzyme inhibitor; ARB, angiotensin receptor blocker; ARNi, angiotensin receptor-neprilysin inhibitor; MRA, mineralocorticoid receptor antagonist; RAASi, renin-angiotensin-aldosterone system inhibitor.

In Germany, Spain, and Sweden, approximately one fifth of patients (17.5%–21.7%) discontinued all RAASi therapy after the hyperkalemia episode; discontinuation was more common in the United Kingdom (37.9%) (Figure 1A and Supplemental Table 2). In Germany, Sweden, and the United Kingdom, 28.4%–47.7% of these patients reinitiated at least one RAASi therapy within 1 year after the hyperkalemia episode; reinitiation was less common in Spain (15.0%) (Figure 1B).

In Germany, Sweden, and the United Kingdom, MRA was the most commonly discontinued RAASi class (35.8%–54.1%). In Spain, ARB was the most commonly discontinued class (34.6%), followed by MRA (14.4%). Reinitiation of MRA within 1 year from the hyperkalemia episode occurred in up to one third (16.9%–32.9%) of patients across all countries.

### Risk of Hospitalizations in Patients with Reduced versus Maintained RAASi

Characteristics of the PS-matched reduced versus maintained RAASi cohorts in each country are presented in Table 2, with the covariates included in the PS matching outlined in the Supplemental Material. SMD plots of covariate balance before and after PS matching are shown in Supplemental Figure 2, and PS distributions are shown in Supplemental Figure 3. In each country, all baseline characteristics that were included as covariates in the PS matching were balanced between the two cohorts after matching (SMD <10%).

**Table 2:** Baseline characteristics of the propensity score–matched reduced versus maintained RAASi cohorts in each country

Characteristic	Germany		Spain		Sweden		United Kingdom	
	Reduced (*n*=3589)	Maintained (*n*=3589)	Reduced (*n*=405)	Maintained (*n*=1048)	Reduced (*n*=5368)	Maintained (*n*=5368)	Reduced (*n*=2642)	Maintained (*n*=2642)
Age, yr, mean (SD)	75.9 (10.7)	75.8 (10.9)	79.5 (8.5)	79.2 (9.5)	76.1 (11.4)	76.0 (11.1)	76.1 (11.8)	76.1 (11.5)
Male, No. (%)	2180 (60.7)	2178 (60.7)	208 (51.4)	531 (50.7)	2270 (42.3)	2253 (42.0)	1487 (56.3)	1505 (57.0)
**Hyperkalemia severity at index,^a^ No. (%)**								
Mild	n/a	n/a	166 (41.0)	408 (38.9)	3327 (62.0)	3495 (65.1)	n/a	n/a
Moderate	n/a	n/a	189 (46.7)	616 (58.8)	1233 (23.0)	1096 (20.4)	n/a	n/a
Severe	n/a	n/a	50 (12.3)	24 (2.3)	808 (15.1)	777 (14.5)	n/a	n/a
**CKD, No. (%)**	2592 (72.2)	2612 (72.8)	361 (89.1)	910 (86.8)	4570 (85.1)	4522 (84.2)	2429 (91.9)	2415 (91.4)
Stage 3	1301 (36.2)	1292 (36.0)	286 (70.6)	696 (66.4)	2962 (55.2)	2954 (55.0)	1410 (53.4)	1420 (53.7)
Stage 4	656 (18.3)	672 (18.7)	53 (13.1)	169 (16.1)	1192 (22.2)	1169 (21.8)	765 (29.0)	734 (27.8)
Stage 5	136 (3.8)	145 (4.0)	10 (2.5)	16 (1.5)	416 (7.8)	399 (7.4)	172 (6.5)	174 (6.6)
Unknown stage	499 (19.3)	503 (19.3)	12 (3.0)	29 (2.8)	0 (0.0)	0 (0.0)	82 (3.1)	87 (3.3)
HF, No. (%)	2512 (70.0)	2498 (69.6)	87 (21.5)	198 (18.9)	3047 (56.8)	3066 (57.1)	799 (30.2)	816 (30.9)
Diabetes, No. (%)	2154 (60.0)	2153 (60.0)	182 (44.9)	466 (44.5)	2429 (45.3)	2457 (45.8)	1494 (56.5)	1486 (56.2)
**RAASi, No. (%)**								
ACEi	1764 (49.2)	1776 (49.5)	98 (24.2)	319 (30.4)	2711 (50.5)	2695 (50.2)	1654 (62.6)	1645 (62.3)
ARB	1277 (35.6)	1311 (36.5)	317 (78.3)	736 (70.2)	2359 (44.0)	2359 (44.0)	837 (31.7)	827 (31.3)
ARNi	261 (7.3)	219 (6.1)	16 (4.0)	39 (3.7)	145 (2.7)	148 (2.8)	33 (1.2)	41 (1.6)
MRA	1198 (33.4)	1167 (32.5)	68 (16.8)	174 (16.6)	1963 (36.6)	1993 (37.1)	602 (22.8)	603 (22.8)

ACEi, angiotensin-converting enzyme inhibitor; ARB, angiotensin receptor blocker; ARNi, angiotensin receptor neprilysin inhibitor; HF, heart failure; MRA, mineralocorticoid receptor antagonist; n/a, not available; RAASi, renin-angiotensin-aldosterone system inhibitor.

a Hyperkalemia severity was defined as follows: mild, >5.0 to <5.5; moderate, 5.5 to <6.0; and severe ≥6.0 mmol/L.

**Figure 2:**
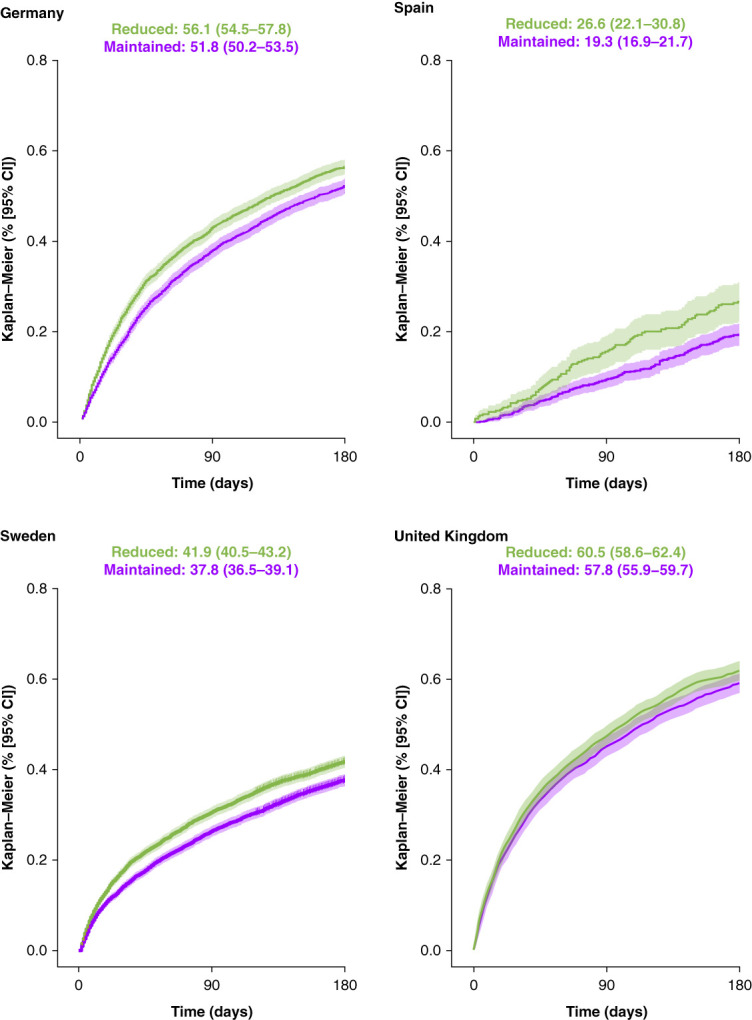
**Risk of a first hospitalization within 6 months in patients with CKD and/or HF who reduced versus maintained RAASi therapy after a hyperkalemia episode.** Six-month cumulative incidences are presented with 95% CI. CI, confidence interval; HF, heart failure.

Across all countries, the 6-month risk of at least one hospitalization was consistently higher in those with reduced versus maintained RAASi therapy (Figure 2). The 6-month cumulative incidences of a first hospitalization were highest in Germany (56.1 reduced RAASi versus 51.8 maintained RAASi) and the United Kingdom (60.5 reduced RAASi versus 57.8 maintained RAASi). The total number of hospitalizations within 6 months of the hyperkalemia episode was also consistently higher in those with reduced RAASi therapy relative to those with maintained RAASi (Table 3).

**Table 3:** Total number of hospitalizations per person-year within 6 months of a hyperkalemia episode in patients with reduced versus maintained RAASi therapy

Country	Total Number of Hospitalizations Per Person-Year (95% CI)	
	Reduced RAASi	Maintained RAASi
Germany	2.45 (2.38 to 2.53)	2.07 (2.01 to 2.14)
Spain	0.87 (0.22 to 3.35)	0.72 (0.27 to 1.88)
Sweden	1.56 (1.49 to 1.62)	1.38 (1.32 to 1.45)
United Kingdom	3.40 (3.30 to 3.50)	2.93 (2.84 to 3.03)

CI, confidence interval; RAASi, renin-angiotensin-aldosterone system inhibitor.

**Figure 3:**
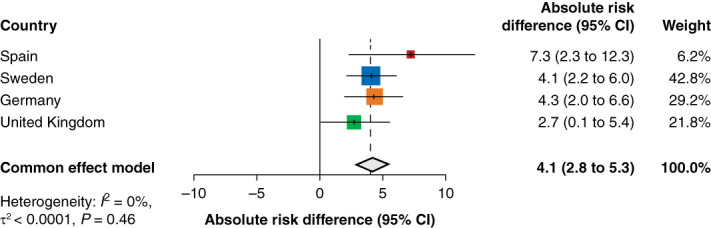
Absolute risk difference in the 6-month risk of at least one hospitalization in patients with CKD and/or HF who maintained versus reduced RAASi therapy after a hyperkalemia episode.

The absolute differences in 6-month risk of a first hospitalization ranged from 2.7% in the United Kingdom to 7.3% in Spain (Figure 3). The meta-analyzed absolute risk difference was 4.1% (95% CI, 2.8 to 5.3). Applying the NNT framework, data suggest that a first hospitalization within 6 months could potentially have been avoided if 25 (95% CI, 19 to 36) patients had maintained instead of reduced their RAASi.

## Discussion

This study, describing routine clinical practice across four distinct European health care systems, showed that RAASi discontinuation after a hyperkalemia episode occurs frequently and persists over time, with fewer than half of patients having reinitiated any RAASi therapy in the year after the hyperkalemia episode. The study also showed that, even within as short a time frame as 6 months, hyperkalemia-related RAASi downtitration or discontinuation was associated with an increased risk of hospitalizations, with an average absolute risk difference of 4.1%. This association was consistently observed across the four different European countries included in the study. This finding is also consistent with those from previous studies, showing an increased risk of adverse cardiorenal outcomes and more inpatient care associated with reduced, compared with maintained, RAASi therapy after a hyperkalemia episode.^15,24^ Applying the NNT framework, our data suggest that a first hospitalization within 6 months could potentially have been avoided if 25 patients had maintained, instead of reduced, their RAASi therapy after a hyperkalemia episode, in alignment with guideline recommendations.^3–5,22^

The absolute risk of a hospitalization within 6 months was highest in Germany and the United Kingdom, which may be a result of differences between countries in patient populations, health care systems, or in how the index hyperkalemia episode was defined. The proportion of patients with CKD at baseline was higher in the United Kingdom (91.6%) compared with the other countries (72.0%–85.2%), whereas the proportion of patients with HF at baseline in Germany was higher (71.3%) than the other countries (17.9%–55.3%). In addition, both Germany and the United Kingdom used hyperkalemia diagnosis codes alone to identify patients with hyperkalemia, while Spain and Sweden also used laboratory results (with the definition of K^+^ >5.0 mmol/L). This may have resulted in a selection of patients with more severe hyperkalemia in Germany and the United Kingdom, for instance, if the threshold for recording a hyperkalemia diagnosis is in some cases higher than K^+^ >5.0 mmol/L. Furthermore, hyperkalemia severity (on the basis of K^+^ values) at the index date was used as a matching covariate in Spain and Sweden, but was thus not possible to account for as a potential baseline confounder in Germany and the United Kingdom. This is a limitation in these countries' analyses because different degrees of hyperkalemia may prompt different management strategies and may also indicate differences in patient risk profiles.

MRA was generally the RAASi class that was most often discontinued, with two thirds of patients or more not reinitiating MRA within a year after the hyperkalemia episode. This aligns with findings from prior studies.^31,32^ MRA significantly reduces the risk of morbidity and mortality in patients with HF and in patients with CKD and diabetes, but because of their inherent high risk of hyperkalemia, close monitoring and management of hyperkalemia is warranted. Furthermore, guidelines state that MRA should be discontinued in patients with persistent hyperkalemia only if it is not possible to manage it with other measures.^3–5,22^ The risk of hyperkalemia may be relatively lower with the newer nonsteroidal MRA finerenone, but its use still warrants careful monitoring of K^+^ levels.^4,33,34^ Sodium-glucose cotransporter 2 inhibitors, which are now the standard of care in both CKD and HF,^3–5^ may reduce the risk of severe hyperkalemia,^35–37^ although their role in hyperkalemia management in clinical practice is still to be determined.^38^

To give clinicians a sense of the magnitude of impact,^29,30^ our study estimated the NNT to avoid a first hospitalization if RAASi therapy had instead been maintained at the prior dose. However, contextualizing NNTs across studies is hampered by differences in study populations, the type of intervention and comparator, outcomes, and the duration of follow-up. Previous studies directly comparable with this study have not been identified. However, NNT has been reported in studies comparing guideline-supported interventions versus placebo or no therapy to avoid a HF-related hospitalization among patients with HF. The NNTs reported in these studies were 41 with the use of MRA (follow-up period ranged between 24 weeks and 3 years),^39^ approximately 21 with the use of *β*-blockers (3-year follow-up period),^40^ 36 with the use of sacubitril valsartan (27-month follow-up period),^41^ and 17–45 (including the risk of cardiovascular death) with sodium-glucose cotransporter 2 inhibitor treatments (follow-up periods varied across studies).^42,43^ Similar studies in patients with CKD have not been identified. To the best of our knowledge, this is the first study of its kind to use the NNT framework to assess the risk of hospitalization associated with reducing and maintaining RAASi therapy after a hyperkalemia episode.

NNT has previously been used as a practical measure to quantify the magnitude of health benefits on the basis of observational studies across disease areas.^44,45^ Because the current NNT was generated from observational data, patients were not randomly allocated to receive reduced or maintained RAASi therapy. Therefore, to balance the cohorts on potential baseline confounders, PS matching was applied, although some residual confounding may remain. However, a previous study within the ZORA study program that applied a similar methodology concluded that there was limited residual confounding as assessed by negative control outcomes.^24^

Limitations in this study include the requirement of at least 120 days of available follow-up time after the index date, which was needed to define whether RAASi therapy was reduced or maintained. This likely led to the selection of a relatively healthier and less frail population by excluding those who died early; thus, the risk of hospitalizations may have been underestimated. However, this requirement was applied equally to both comparison cohorts. Furthermore, the identification of patients with HF or CKD at baseline was predominantly based on ICD-10 diagnosis codes, although baseline eGFR measurements were available in some data sources; therefore, differentiation between patients with HF with preserved or reduced ejection fraction was not possible. Nevertheless, the analytical approach applied in this study, which assessed a reduction of the patients' prehyperkalemia RAASi therapy rather than attaining the maximum dose of specific RAASi classes, was thus not sensitive to how different guidelines vary in their recommendations on specific RAASi classes. Furthermore, in some instances, factors other than hyperkalemia may have affected RAASi therapy use, such as low BP or AKI. However, both limitations affected the two cohorts equally. Given the exposure assessment window for categorizing patients as having reduced or maintained RAASi therapy overlapped with the outcome assessment window, there may have been risk of time-related bias. The rationale for this design element was that changes in RAASi regimen are likely implemented at or soon after the hyperkalemia episode, but the exposure categorization must allow time for patients to collect a new prescription (or not). Furthermore, defining RAASi reduction as either downtitration or discontinuation was based on a previous study showing similarly elevated risks of adverse outcomes with each of these strategies.^15^ Finally, the outcome all-cause hospitalizations is of importance to both patients and health care systems, but its broadness may have reduced specificity and diluted the association. Given that these datasets capture coded admissions rather than adjudicated clinical end points, identifying hospitalizations as related to specific conditions that may be exacerbated by reduced RAASi therapy, particularly in an already vulnerable population,^46,47^ would rely on diagnostic coding practices, as well as data availability and granularity, which differ between countries and may introduce even further uncertainties. All-cause hospitalizations could also be assessed equally in all countries, although it could still be impacted to some degree by country-specific health care systems.

Notable additional strengths were the inclusion of large numbers of patients from routine clinical practice across four different European countries. The increased hospitalization risk associated with hyperkalemia-related RAASi reduction was consistently observed across all included countries, despite country-level differences in how the index hyperkalemia episode was defined (using hyperkalemia diagnosis codes only or also using K^+^ values), in how baseline CKD was defined (by CKD diagnosis only or also by eGFR), as well as in the baseline characteristics of included patients (*e.g*., baseline HF and MRA use were less common in Spain than in other countries).

Across four different European countries, downtitration or discontinuation of RAASi therapy after a hyperkalemia episode occurred frequently and persisted over time among patients with CKD and/or HF. Relative to maintained RAASi therapy, a reduction was consistently associated with an increased risk of hospitalizations. Applying the NNT framework, this study suggests that by increasing adherence to guideline recommendations, a first hospitalization within 6 months could potentially have been avoided if 25 patients had maintained instead of reduced RAASi therapy after a hyperkalemia episode.

## Supplementary Material

## Data Availability

Data underlying the findings described in this manuscript may be obtained *via* the corresponding author upon reasonable request, in accordance with AstraZeneca's data sharing policy described at https://astrazenecagrouptrials.pharmacm.com/ST/Submission/Disclosure. However, restrictions apply to these data, which were used under license and/or specific approvals for the current study and are not publicly available.

## Supplemental Material

Data Sources

Reduced and Maintained Renin-Angiotensin-Aldosterone System Inhibitor (RAASi) Definitions

Covariates Evaluated for Inclusion in Propensity Score (PS) Matching

Supplemental Table 1. Baseline characteristics of patients with reduced versus maintained RAASi before propensity score matching.

Supplemental Table 2. RAASi discontinuation after an index hyperkalemia episode (with patient numbers).

Supplemental Figure 1. Patient attrition in (A) Germany, (B) Spain, (C) Sweden, and (D) the United Kingdom.

Supplemental Figure 2. SMD plots of covariate balance before and after propensity score matching in (A) Germany, (B) Spain, (C) Sweden, and (D) the United Kingdom.

Supplemental Figure 3. Distribution of propensity scores before and after matching in (A) Germany, (B) Spain, (C) Sweden, and (D) the United Kingdom.
