# Correction to: Estimated Number Needed to Treat to Avoid a First Hospitalization by Maintaining Instead of Reducing Renin-Angiotensin-Aldosterone System Inhibitor (RAASi) Therapy after Hyperkalemia

**DOI:** 10.34067/KID.0000000980

**Published:** 2025-08-26

**Authors:** Maria K. Svensson, Michael Fischereder, Paul R. Kalra, Ignacio José Sánchez Lázaro, Eva Lesén, Stefan Franzén, Alaster Allum, Thomas Cars, Nils Kossack, Philipp Breitbart, David Arroyo

In the study by Svensson *et al*, “Estimated Number Needed to Treat to Avoid a First Hospitalization by Maintaining Instead of Reducing Renin-Angiotensin-Aldosterone System Inhibitor (RAASi) Therapy after Hyperkalemia” (*Kidney360* 5(12): 1813–1823. doi: 10.34067/KID.0000000000000561), corrections are needed.

The authors note that the Acknowledgments section should be listed as the following:

The authors would like to thank Marc Pignot and Nikolaus Kolb (ZEG—Berlin Center for Epidemiology and Health Research GmbH, Berlin, Germany) for supervision and project administration for the German analyses; Meg Parbrook and Clement Erhard (AstraZeneca, Cambridge, the United Kingdom) for the UK data analyses; and Ignacio Hernández (Atrys Health, Madrid, Spain) for the Spain data analyses.

This study is based in part on data from the Clinical Practice Research Datalink obtained under license from the United Kingdom Medicines and Healthcare products Regulatory Agency (study protocol 23_003219). The data are provided by patients and collected by the National Health Service as part of their care and support. The interpretation and conclusions contained in this study are those of the authors alone. Office for National Statistics (ONS) data are provided by ONS. ONS and Hospital Episode Statistics data are copyright (c) 2025, reused with the permission of The Health & Social Care Information Centre. All rights reserved.

Medical writing support was provided by Nathan Andrade, MSc, and editorial support was provided by Jess Fawcett, BSc, both of Core (a division of Prime, London, the United Kingdom), supported by AstraZeneca according to Good Publication Practice guidelines (https://www.acpjournals.org/doi/epdf/10.7326/M22-1460). AstraZeneca was involved in the study design, collection, analysis, and interpretation of data. However, ultimate responsibility for opinions, conclusions, and data interpretation lies with the authors.

