# Supplementary material for: Estimated Number Needed to Treat to Avoid a First Hospitalization by Maintaining Instead of Reducing Renin-Angiotensin-Aldosterone System Inhibitor (RAASi) Therapy after Hyperkalemia
Source: Kidney360. 2024 Aug 21;5(12):1813–23. doi: 10.34067/KID.0000000000000561 (PMC11687985; doi:10.34067/KID.0000000000000561)
Supplement: Supplementary file 1 [file kidney360-5-1813-s001.pdf]

ASN American Society of Nephrology
JASN Journal of the American Society of Nephrology
KIDNEY360 Accessing Our World From Every Angle
CJASN Clinical Journal of the American Society of Nephrology

ASN Journal Disclosure Form

As per ASN journal policy, I have disclosed any financial relationships or commitments I have held in the past 36 months as included below. I have listed my Current Employer below to indicate there is a relationship requiring disclosure. If no relationship exists, my Current Employer is not listed.

A. Allum reports the following:
Employer: AstraZeneca; and Ownership Interest: AstraZeneca.

I understand that the information above will be published within the journal article, if accepted, and that failure to comply and/or to accurately and completely report the potential financial conflicts of interest could lead to the following: 1) Prior to publication, article rejection, or 2) Post-publication, sanctions ranging from, but not limited to, issuing a correction, reporting the inaccurate information to the authors' institution, banning authors from submitting work to ASN journals for varying lengths of time, and/or retraction of the published work.

Name: Alaster Allum
Manuscript ID: K360-2024-000349R1
Manuscript Title: Hyperkalemia-Related RAASi Reduction and Estimated Number Needed to Treat to Avoid a First Hospitalization by Maintaining RAASi
Date of Completion: August 13, 2024
Disclosure Updated Date: May 21, 2024

ASN
American Society of Nephrology
JASN
Journal of the American Society of Nephrology
KIDNEY360
Accessing Our World From Every Angle
CJASN
Clinical Journal of the American Society of Nephrology

ASN Journal Disclosure Form

As per ASN journal policy, I have disclosed any financial relationships or commitments I have held in the past 36 months as included below. I have listed my Current Employer below to indicate there is a relationship requiring disclosure. If no relationship exists, my Current Employer is not listed.

D. Arroyo reports the following:
Employer: Hospital General Universitario Gregorio Marañón; Honoraria: Abbot, AstraZeneca, Bayer, Boehringer Ingelheim, CSL Vifor, Eli-Lilly, Esteve, GSK, Menarini, Novo Nordisk, Otsuka; and Advisory or Leadership Role: Bayer.

I understand that the information above will be published within the journal article, if accepted, and that failure to comply and/or to accurately and completely report the potential financial conflicts of interest could lead to the following: 1) Prior to publication, article rejection, or 2) Post-publication, sanctions ranging from, but not limited to, issuing a correction, reporting the inaccurate information to the authors' institution, banning authors from submitting work to ASN journals for varying lengths of time, and/or retraction of the published work.

Name: David A. Arroyo
Manuscript ID: K360-2024-000349R1
Manuscript Title: Hyperkalemia-Related RAASi Reduction and Estimated Number Needed to Treat to Avoid a First Hospitalization by Maintaining RAASi
Date of Completion: July 7, 2024
Disclosure Updated Date: July 7, 2024

ASN American Society of Nephrology | JASN Journal of the American Society of Nephrology | KIDNEY360 Accessing Our World From Every Angle | CJASN Clinical Journal of the American Society of Nephrology

ASN Journal Disclosure Form

As per ASN journal policy, I have disclosed any financial relationships or commitments I have held in the past 36 months as included below. I have listed my Current Employer below to indicate there is a relationship requiring disclosure. If no relationship exists, my Current Employer is not listed.

P. Breitbart reports the following:
Employer: University Heart Center Freiburg - Bad Krozingen; Consultancy: Speaker honoraria and consultancy fees from AstraZeneca, Bayer Vital, Bristol-Myers Squibb, Daiichi Sankyo, Novo Nordisk, Pfizer, and Sanofi Aventis.; Research Funding: Deutsche Herzstiftung e.V.; and Honoraria: Speaker honoraria and consultancy fees from AstraZeneca, Bayer Vital, Bristol-Myers Squibb, Daiichi Sankyo, Novo Nordisk, Pfizer, and Sanofi Aventis.

I understand that the information above will be published within the journal article, if accepted, and that failure to comply and/or to accurately and completely report the potential financial conflicts of interest could lead to the following: 1) Prior to publication, article rejection, or 2) Post-publication, sanctions ranging from, but not limited to, issuing a correction, reporting the inaccurate information to the authors' institution, banning authors from submitting work to ASN journals for varying lengths of time, and/or retraction of the published work.

Name: Philipp Breitbart
Manuscript ID: K360-2024-000349R1
Manuscript Title: Hyperkalemia-Related RAASi Reduction and Estimated Number Needed to Treat to Avoid a First Hospitalization by Maintaining RAASi
Date of Completion: July 12, 2024
Disclosure Updated Date: July 12, 2024

ASN
American Society of Nephrology
JASN
Journal of the American Society of Nephrology
KIDNEY360
Accessing Our World From Every Angle
CJASN
Clinical Journal of the American Society of Nephrology

ASN Journal Disclosure Form

As per ASN journal policy, I have disclosed any financial relationships or commitments I have held in the past 36 months as included below. I have listed my Current Employer below to indicate there is a relationship requiring disclosure. If no relationship exists, my Current Employer is not listed.

T. Cars reports the following:
Employer: Sence Research AB; Consultancy: Thomas Cars is an employee and shareholder in Sence Research AB (Sence Research AB, a company in epidemiology and biostatistics with both healthcare authorities and private companies as clients); Ownership Interest: Sence Research AB; Ctatistics AB; Guard Therapeutics; Camurus; Research Funding: Thomas Cars is an employee and shareholder in Sence Research AB (Sence Research AB, a company in epidemiology and biostatistics with both healthcare authorities and private companies as clients); and Advisory or Leadership Role: CEO at Sence Research AB.

I understand that the information above will be published within the journal article, if accepted, and that failure to comply and/or to accurately and completely report the potential financial conflicts of interest could lead to the following: 1) Prior to publication, article rejection, or 2) Post-publication, sanctions ranging from, but not limited to, issuing a correction, reporting the inaccurate information to the authors' institution, banning authors from submitting work to ASN journals for varying lengths of time, and/or retraction of the published work.

Name: Thomas Cars
Manuscript ID: K360-2024-000349R1
Manuscript Title: Hyperkalemia-Related RAASi Reduction and Estimated Number Needed to Treat to Avoid a First Hospitalization by Maintaining RAASi,
Date of Completion: July 9, 2024
Disclosure Updated Date: July 9, 2024

ASN American Society of Nephrology | JASN Journal of the American Society of Nephrology | KIDNEY360 Accessing Our World From Every Angle | CJASN Clinical Journal of the American Society of Nephrology

ASN Journal Disclosure Form

As per ASN journal policy, I have disclosed any financial relationships or commitments I have held in the past 36 months as included below. I have listed my Current Employer below to indicate there is a relationship requiring disclosure. If no relationship exists, my Current Employer is not listed.

M. Fischereder reports the following:
Employer: Klinikum der LMU; Kuratorium fuer Dialyse und Nierentransplantation; Consultancy: Thermosome; Novartis; Sanofi; Ownership Interest: Novartis (share holder); Research Funding: Novartis, AstraZeneca; Honoraria: Astellas, AstraZeneca, Bayer Vital, Boehringer, Novartis, Pfizer, Sanofi, Vifor; Advisory or Leadership Role: Eurotransplant Kidney Advisory Committee (ETKAC, chair, not paid service); Speakers Bureau: Astellas, AstraZeneca, Bayer Vital, Boehringer, Novartis, Pfizer, Sanofi, Vifor; and Other Interests or Relationships: board member: KfH-Stiftung.

I understand that the information above will be published within the journal article, if accepted, and that failure to comply and/or to accurately and completely report the potential financial conflicts of interest could lead to the following: 1) Prior to publication, article rejection, or 2) Post-publication, sanctions ranging from, but not limited to, issuing a correction, reporting the inaccurate information to the authors' institution, banning authors from submitting work to ASN journals for varying lengths of time, and/or retraction of the published work.

Name: Michael Fischereder
Manuscript ID: K360-2024-000349R1
Manuscript Title: Hyperkalemia-Related RAASi Reduction and Estimated Number Needed to Treat to Avoid a First Hospitalization by Maintaining RAASi
Date of Completion: August 16, 2024
Disclosure Updated Date: August 16, 2024

ASN American Society of Nephrology | JASN Journal of the American Society of Nephrology | KIDNEY360 Accessing Our World From Every Angle | CJASN Clinical Journal of the American Society of Nephrology

ASN Journal Disclosure Form

As per ASN journal policy, I have disclosed any financial relationships or commitments I have held in the past 36 months as included below. I have listed my Current Employer below to indicate there is a relationship requiring disclosure. If no relationship exists, my Current Employer is not listed.

S. Franzén reports the following:
Employer: AstraZeneca; and Ownership Interest: AstraZeneca.

I understand that the information above will be published within the journal article, if accepted, and that failure to comply and/or to accurately and completely report the potential financial conflicts of interest could lead to the following: 1) Prior to publication, article rejection, or 2) Post-publication, sanctions ranging from, but not limited to, issuing a correction, reporting the inaccurate information to the authors' institution, banning authors from submitting work to ASN journals for varying lengths of time, and/or retraction of the published work.

Name: Stefan Franzén
Manuscript ID: K360-2024-000349R1
Manuscript Title: Hyperkalemia-Related RAASi Reduction and Estimated Number Needed to Treat to Avoid a First Hospitalization by Maintaining RAASi
Date of Completion: August 5, 2024
Disclosure Updated Date: August 5, 2024

ASN Journal Disclosure Form

As per ASN journal policy, I have disclosed any financial relationships or commitments I have held in the past 36 months as included below. I have listed my Current Employer below to indicate there is a relationship requiring disclosure. If no relationship exists, my Current Employer is not listed.

P. Kalra reports the following:
Employer: Portsmouth Hospitals University NHS Trust; Ownership Interest: Portsmouth Cardiac Associates; Hampshire Heart Clinic; Research Funding: Pharmacosmos; FIRE-1;; Honoraria: Amgen; Boehringer Ingelheim; Pharmacosmos; CSL Vifor; and Speakers Bureau: AstraZeneca; Bayer; Pharmacosmos; CSL Vifor; Amgen; Novartis; Pfizer.

I understand that the information above will be published within the journal article, if accepted, and that failure to comply and/or to accurately and completely report the potential financial conflicts of interest could lead to the following: 1) Prior to publication, article rejection, or 2) Post-publication, sanctions ranging from, but not limited to, issuing a correction, reporting the inaccurate information to the authors' institution, banning authors from submitting work to ASN journals for varying lengths of time, and/or retraction of the published work.

Name: Paul Kalra
Manuscript ID: K360-2024-000349R1
Manuscript Title: Hyperkalemia-Related RAASi Reduction and Estimated Number Needed to Treat to Avoid a First Hospitalization by Maintaining RAASi
Date of Completion: August 14, 2024
Disclosure Updated Date: August 14, 2024

ASN Journal Disclosure Form

As per ASN journal policy, I have disclosed any financial relationships or commitments I have held in the past 36 months as included below. I have listed my Current Employer below to indicate there is a relationship requiring disclosure. If no relationship exists, my Current Employer is not listed.

N. Kossack reports the following:
Employer: Wissenschaftliches Institut für Gesundheitsökonomie und Gesundheitssystemforschung

I understand that the information above will be published within the journal article, if accepted, and that failure to comply and/or to accurately and completely report the potential financial conflicts of interest could lead to the following: 1) Prior to publication, article rejection, or 2) Post-publication, sanctions ranging from, but not limited to, issuing a correction, reporting the inaccurate information to the authors' institution, banning authors from submitting work to ASN journals for varying lengths of time, and/or retraction of the published work.

Name: Nils Kossack
Manuscript ID: K360-2024-000349R1
Manuscript Title: Hyperkalemia-Related RAASi Reduction and Estimated Number Needed to Treat to Avoid a First Hospitalization by Maintaining RAASi
Date of Completion: August 10, 2024
Disclosure Updated Date: August 10, 2024

ASN American Society of Nephrology
JASN Journal of the American Society of Nephrology
KIDNEY360 Accessing Our World From Every Angle
CJASN Clinical Journal of the American Society of Nephrology

ASN Journal Disclosure Form

As per ASN journal policy, I have disclosed any financial relationships or commitments I have held in the past 36 months as included below. I have listed my Current Employer below to indicate there is a relationship requiring disclosure. If no relationship exists, my Current Employer is not listed.

E. Lesén reports the following:
Employer: AstraZeneca; and Ownership Interest: AstraZeneca.

I understand that the information above will be published within the journal article, if accepted, and that failure to comply and/or to accurately and completely report the potential financial conflicts of interest could lead to the following: 1) Prior to publication, article rejection, or 2) Post-publication, sanctions ranging from, but not limited to, issuing a correction, reporting the inaccurate information to the authors' institution, banning authors from submitting work to ASN journals for varying lengths of time, and/or retraction of the published work.

Name: Eva Lesén
Manuscript ID: K360-2024-000349R1
Manuscript Title: Hyperkalemia-Related RAASi Reduction and Estimated Number Needed to Treat to Avoid a First Hospitalization by Maintaining RAASi
Date of Completion: July 22, 2024
Disclosure Updated Date: July 22, 2024

ASN American Society of Nephrology | JASN Journal of the American Society of Nephrology | KIDNEY360 Accessing Our World From Every Angle | CJASN Clinical Journal of the American Society of Nephrology

ASN Journal Disclosure Form

As per ASN journal policy, I have disclosed any financial relationships or commitments I have held in the past 36 months as included below. I have listed my Current Employer below to indicate there is a relationship requiring disclosure. If no relationship exists, my Current Employer is not listed.

I. Sanchez-Lazaro reports the following:
Employer: Hospital Vithas 9 de Octubre; and Speakers Bureau: Astra Zeneca; Novartis;.

I understand that the information above will be published within the journal article, if accepted, and that failure to comply and/or to accurately and completely report the potential financial conflicts of interest could lead to the following: 1) Prior to publication, article rejection, or 2) Post-publication, sanctions ranging from, but not limited to, issuing a correction, reporting the inaccurate information to the authors' institution, banning authors from submitting work to ASN journals for varying lengths of time, and/or retraction of the published work.

Name: Ignacio J Sanchez-Lazaro
Manuscript ID: K360-2024-000349R1
Manuscript Title: Hyperkalemia-Related RAASi Reduction and Estimated Number Needed to Treat to Avoid a First Hospitalization by Maintaining RAASi,
Date of Completion: July 7, 2024
Disclosure Updated Date: July 7, 2024

ASN
American Society of Nephrology
JASN
Journal of the American Society of Nephrology
KIDNEY360
Accessing Our World From Every Angle
CJASN
Clinical Journal of the American Society of Nephrology

ASN Journal Disclosure Form

As per ASN journal policy, I have disclosed any financial relationships or commitments I have held in the past 36 months as included below. I have listed my Current Employer below to indicate there is a relationship requiring disclosure. If no relationship exists, my Current Employer is not listed.

M. Svensson reports the following:
Employer: Uppsala University, Uppsala, Sweden; and Honoraria: Amgen, AstraZeneca, Boehringer Ingelheim, GSK, NovoNordisk.

I understand that the information above will be published within the journal article, if accepted, and that failure to comply and/or to accurately and completely report the potential financial conflicts of interest could lead to the following: 1) Prior to publication, article rejection, or 2) Post-publication, sanctions ranging from, but not limited to, issuing a correction, reporting the inaccurate information to the authors' institution, banning authors from submitting work to ASN journals for varying lengths of time, and/or retraction of the published work.

Name: Maria K. Svensson
Manuscript ID: K360-2024-000349R1
Manuscript Title: Hyperkalemia-Related RAASi Reduction and Estimated Number Needed to Treat to Avoid a First Hospitalization by Maintaining RAASi
Date of Completion: July 4, 2024
Disclosure Updated Date: May 11, 2024
