# Supplementary material for: Estimated Number Needed to Treat to Avoid a First Hospitalization by Maintaining Instead of Reducing Renin-Angiotensin-Aldosterone System Inhibitor (RAASi) Therapy after Hyperkalemia
Source: Kidney360. 2024 Aug 21;5(12):1813–23. doi: 10.34067/KID.0000000000000561 (PMC11687985; doi:10.34067/KID.0000000000000561)
Supplement: Supplementary file 2 [file kidney360-5-1813-s002.pdf]

Supplemental Material:

Hyperkalemia-Related RAASi Reduction and Estimated Number Needed to Treat to Avoid a First Hospitalization by Maintaining RAASi in Patients with Chronic Kidney Disease and/or Heart Failure

Maria K. Svensson,[1,2] Michael Fischereder,[3] Paul R. Kalra,[4] Ignacio José Sánchez Lázaro,[5] Eva Lesén,[6] Stefan Franzén,[7] Alaster Allum,[8] Thomas Cars,[9] Nils Kossack,[10] Philipp Breitbart,[11] David Arroyo[12]

[1]Department of Medical Sciences, Renal Medicine, Uppsala University, Uppsala, Sweden; [2]Uppsala Clinical Research Centre, Uppsala University, Uppsala, Sweden; [3]Division of Nephrology, Medizinische Klinik und Poliklinik IV, LMU Klinikum, LMU Munich, Munich, Germany; [4]Department of Cardiology, Portsmouth Hospitals University NHS Trust, Portsmouth, UK; [5]Cardiology Department, Hospital Universitari i Politècnic La Fe, Valencia, Spain; [6]CVRM Evidence Strategy, BioPharmaceuticals Medical, AstraZeneca, Gothenburg, Sweden; [7]Medical & Payer Evidence Statistics, AstraZeneca, Gothenburg, Sweden; [8]BioPharmaceuticals Medical CVRM, AstraZeneca, Cambridge, UK; [9]Sence Research AB, Uppsala, Sweden; [10]WIG2 Scientific Institute for Health Economics and Health System Research, Leipzig, Germany; [11]Department of Cardiology and Angiology, Medical Center, Faculty of Medicine, University of Freiburg, Bad Krozingen, Germany; [12]Nephrology Department, Hospital General Universitario Gregorio Marañón, Madrid, Spain

Corresponding author: Maria K. Svensson

Address: Department of Medical Sciences, Renal Medicine, Uppsala University, Akademiska sjukhuset, Entrance 40, floor 5, SE 751 85 Uppsala, Sweden;

Phone: +46(0)722 121719; **e-mail:** maria.k.svensson@medsci.uu.se

Table of Contents

Data Sources 3

Covariates Evaluated for Inclusion in Propensity Score (PS) Matching 4

Supplemental Table 1. Baseline characteristics of patients with reduced versus maintained RAASi before propensity score matching 5

Supplemental Table 2. RAASi discontinuation after an index hyperkalemia episode (with patient numbers) 7

Supplemental Figure 1. Patient attrition in (A) Germany, (B) Spain, (C) Sweden, and (D) the UK 9

Supplemental Figure 2. SMD plots of covariate balance before and after propensity score matching in (A) Germany, (B) Spain, (C) Sweden, and (D) the UK 11

Supplemental Figure 3. Distribution of propensity scores before and after matching in (A) Germany, (B) Spain, (C) Sweden, and (D) the UK 13

References 17

Data Sources

In Germany, the WIG2 benchmark healthcare claims database contains longitudinal, routinely collected, and anonymized administrative data from about 4.5 million patients insured by the German statutory health insurance from January 1, 2014 through December 31, 2022.[1] The WIG2 database provides a representative sample (in terms of age, sex, and morbidity) of the German population. Data include recorded diagnoses and procedures in outpatient and inpatient care settings, prescriptions, date of death and end of insurance coverage (for censoring purposes). The study period was January 2018−December 2022.

The Spanish BIG-PAC administrative database includes anonymized electronic medical records data for nearly 2 million patients from primary and secondary care within the Spanish national health system and across seven Spanish regions.[2] The study period was July 2021−December 2022.

The Swedish database includes regional data on laboratory and clinical measurements from electronic health records in two of the largest regions in Sweden (Region Stockholm and Region Skåne), linked with three national Swedish registries: the Prescribed Drug Register (with data on filled prescriptions), the National Patient Registry (covering diagnoses and procedures recorded in inpatient and outpatient care settings), and the Cause of Death Registry. The data sources are linked via unique personal identification numbers. The study period was March 2018−July 2020.

The UK Clinical Practice Research Datalink (CPRD) Aurum database includes primary care data collected from general practices across the UK,[3] capturing

diagnoses, issued prescriptions, and laboratory tests. CPRD Aurum was linked to the Hospital Episode Statistics (HES) for data on secondary care, and to the Office for National Statistics (ONS) for data on death registrations. The study period was January 2018−March 2021.

Covariates Evaluated for Inclusion in Propensity Score (PS) Matching

The following covariates were evaluated for inclusion in the PS matching: demographics (age and sex), comorbidities (chronic kidney disease [CKD] overall and by stage, arrhythmia, heart failure [HF], coronary heart disease, diabetes, proteinuria, comorbidity index), comedications (RAASi, alpha blockers, beta blockers, beta agonists, cardiac glycosides, calcium channel blockers, calcium gluconate, diuretics [any], loop diuretics, thiazide diuretics, insulin, sodium bicarbonate, non-steroidal anti-inflammatory drugs, and sodium-glucose co-transporter-2 inhibitors, and total number of different medication classes based on four-level Anatomical Therapeutic Chemical codes), healthcare resource use (inpatient bed-days, outpatient visits, emergency department visits), potassium value at index, history of potassium binder use, and estimated glomerular filtration rate.

Supplemental Table 1. Baseline characteristics of patients with reduced versus maintained RAASi before propensity score matching

	Germany		Spain		Sweden		UK	
	Reduced (*N*=5185)	Maintained (*N*=6492)	Reduced (*N*=1089)	Maintained (*N*=3303)	Reduced (*N*=6036)	Maintained (*N*=11,368)	Reduced (*N*=3760)	Maintained (*N*=2826)
Age, mean (SD), yr	76.0 (10.5)	75.7 (10.8)	79.1 (9.2)	78.4 (9.7)	76.0 (11.3)	75.5 (11.6)	76.7 (11.6)	75.7 (11.7)
Male, *n* (%)	3135 (60.5)	3941 (60.7)	556 (51.1)	1675 (50.7)	2537 (42.0)	4791 (42.1)	2086 (55.5)	1612 (57.0)
Hyperkalemia severity at index,[a] *n* (%)								
Mild	n/a	n/a	168 (15.4)	2046 (61.9)	3620 (60.0)	7907 (69.6)	n/a	n/a
Moderate	n/a	n/a	412 (37.8)	1230 (37.2)	1360 (22.5)	2066 (18.2)	n/a	n/a
Severe	n/a	n/a	509 (46.7)	27 (0.8)	980 (16.2)	1261 (11.1)	n/a	n/a
CKD, *n* (%)	3662 (70.6)	4740 (73.0)	952 (87.4)	2790 (84.5)	5160 (85.5)	9247 (81.3)	3484 (92.7)	2551 (90.3)
Stage 3	1864 (35.9)	2470 (38.0)	747 (68.6)	2247 (68.0)	3246 (53.8)	6692 (58.9)	1811 (48.2)	1529 (54.1)
Stage 4	929 (17.9)	1028 (15.8)	147 (13.5)	411 (12.4)	1417 (23.5)	2010 (17.7)	1223 (32.5)	742 (26.3)
Stage 5	168 (3.2)	237 (3.7)	29 (2.7)	55 (1.7)	497 (8.2)	545 (4.8)	343 (9.1)	175 (6.2)
Unknown stage	701 (13.5)	1005 (15.5)	29 (2.7)	77 (2.3)	0 (0.0)	0 (0.0)	107 (2.8)	105 (3.7)

HF, *n* (%)	3873 (74.7)	4452 (68.6)	287 (26.4)	499 (15.1)	3539 (58.6)	6083 (53.5)	1222 (32.5)	899 (31.8)
Diabetes, *n* (%)	3096 (59.7)	4037 (62.2)	516 (47.4)	1397 (42.3)	2712 (44.9)	5377 (47.3)	2082 (55.4)	1580 (55.9)
RAASi, *n* (%)								
ACEi	2664 (51.4)	3122 (48.1)	194 (17.8)	1702 (51.5)	3104 (51.4)	5498 (48.4)	2445 (65.0)	1745 (61.7)
ARB	1995 (38.5)	2465 (38.0)	936 (86.0)	1486 (45.0)	2708 (44.9)	4939 (43.5)	1138 (30.3)	888 (31.4)
ARNi	410 (7.9)	428 (6.6)	36 (3.3)	174 (5.3)	238 (3.9)	256 (2.3)	43 (1.1)	54 (1.9)
MRA	2706 (52.2)	1370 (21.1)	134 (12.3)	561 (17.0)	2475 (41.0)	2792 (24.6)	1186 (31.5)	625 (22.1)

ACEi, angiotensin-converting enzyme inhibitor; ARB, angiotensin receptor blocker; ARNi, angiotensin receptor-neprilysin inhibitor; CKD, chronic kidney disease; HF, heart failure; MRA, mineralocorticoid receptor antagonist; n, number of patients; n/a, not applicable; RAASi, renin-angiotensin-aldosterone system inhibitor; SD, standard deviation.

[a]Hyperkalemia severity was defined as follows: mild, >5.0–<5.5; moderate, 5.5–<6.0; severe ≥6.0 mmol/l.

Supplemental Table 2. RAASi discontinuation after an index hyperkalemia episode (with patient numbers)

		Pre-index, *N*	Discontinued, *n* (%)	With 365-day follow-up, *N*	Reinitiated, *n* (%)
ACEi	Germany	5786	1928 (33.3)	1324	431 (32.6)
	Spain	1896	150 (7.9)	87	19 (21.8)
	Sweden	8602	2034 (23.6)	1249	371 (29.7)
	UK	4190	1695 (40.5)	1043	184 (17.6)
ARB	Germany	4460	1295 (29.0)	845	269 (31.8)
	Spain	2422	838 (34.6)	693	97 (14.0)
	Sweden	7647	1670 (21.8)	1029	421 (40.9)
	UK	2026	820 (40.5)	502	76 (15.1)
ARNi	Germany	838	189 (22.6)	103	44 (42.7)
	Spain	210	9 (4.3)	n/a	n/a
	Sweden	494	77 (15.6)	45	11 (24.4)
	UK	97	20 (20.6)	10	0 (0.0)
MRA	Germany	4076	2204 (54.1)	1512	439 (29.0)
	Spain	695	100 (14.4)	26	7 (26.9)

	Sweden	5267	1886 (35.8)	1179	388 (32.9)
	UK	1811	855 (47.2)	514	87 (16.9)
All RAASi	Germany	11,677	2536 (21.7)	1684	803 (47.7)
	Spain	4392	939 (21.4)	781	117 (15.0)
	Sweden	17,404	3039 (17.5)	1817	815 (44.9)
	UK	6586	2497 (37.9)	1530	434 (28.4)

ACEi, angiotensin-converting enzyme inhibitors; ARB, angiotensin-receptor blockers; ARNi, angiotensin receptor neprilysin inhibitor; MRA, mineralocorticoid receptor antagonists; n/a, not applicable; RAASi, renin-angiotensin-aldosterone inhibitor.

Supplemental Figure 1. Patient attrition in (A) Germany, (B) Spain, (C) Sweden, and (D) the UK

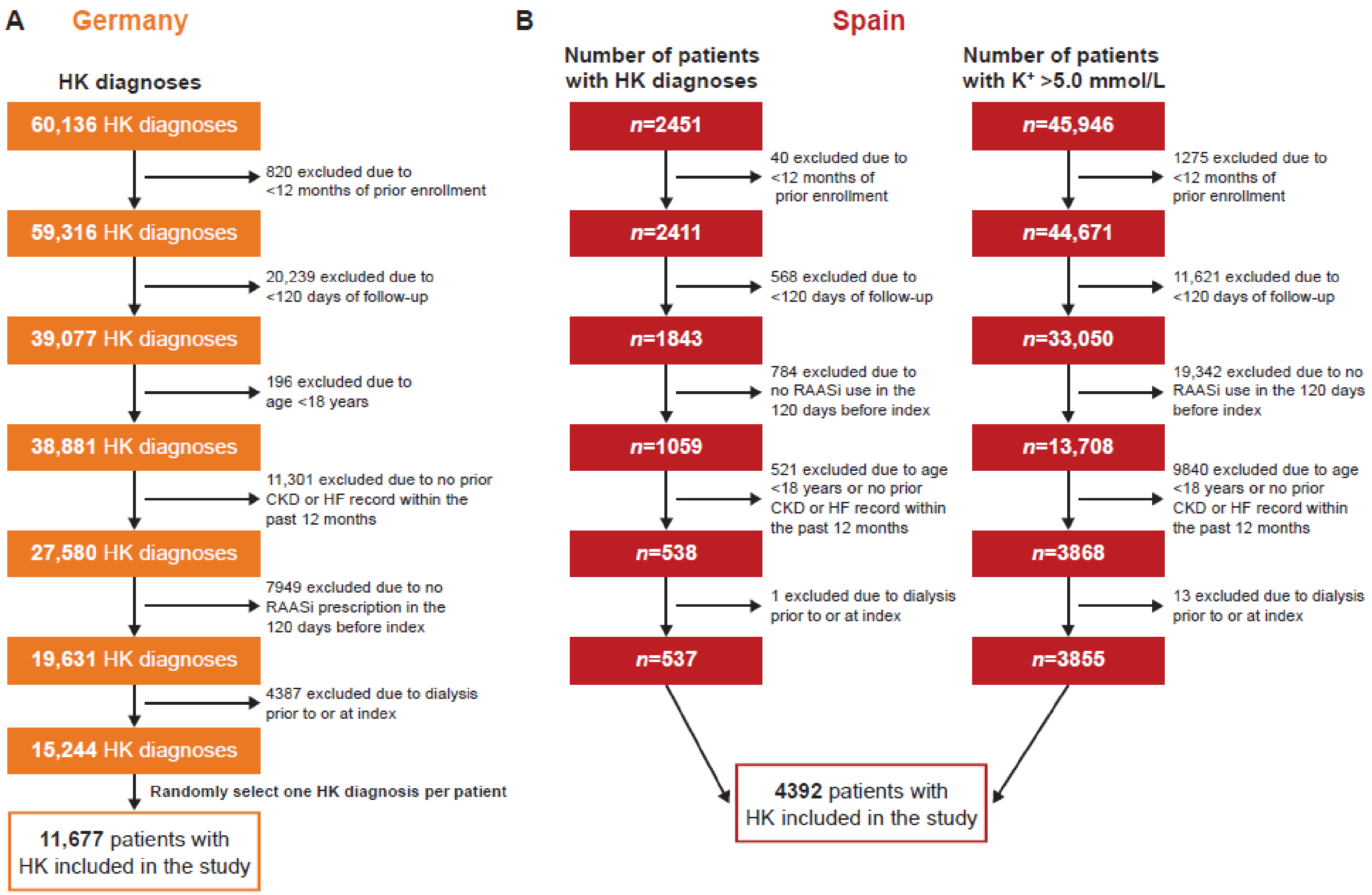

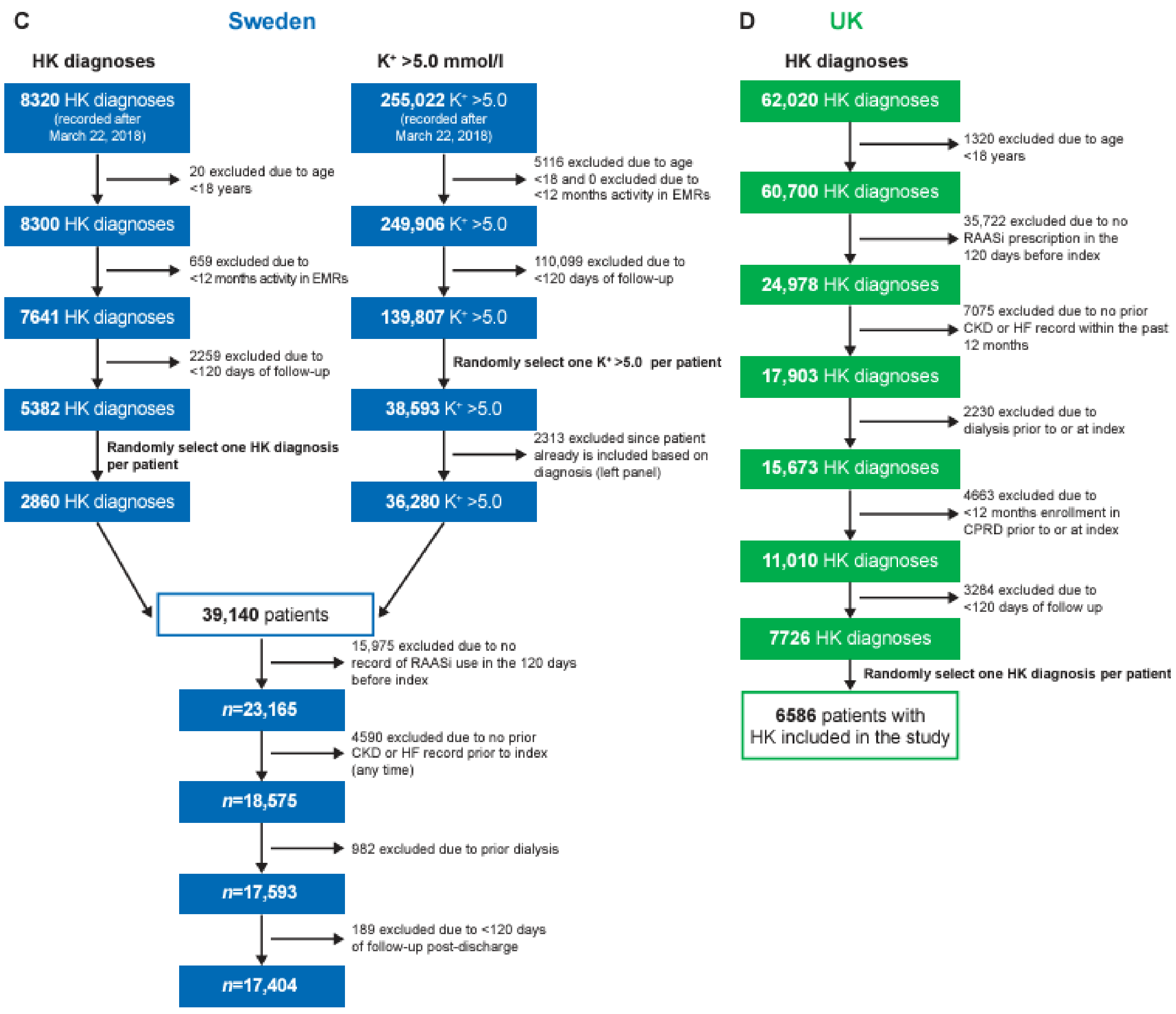

CKD, chronic kidney disease; CPRD, Clinical Practice Research Datalink; Dx, diagnosis; EMR, electronic medical record; FU, follow-up; HF, heart failure; HK, hyperkalemia; K^+, potassium; RAASi, renin-angiotensin-aldosterone system inhibitor.

Supplemental Figure 2. SMD plots of covariate balance before and after propensity score matching in (A) Germany, (B) Spain, (C) Sweden, and (D) the UK

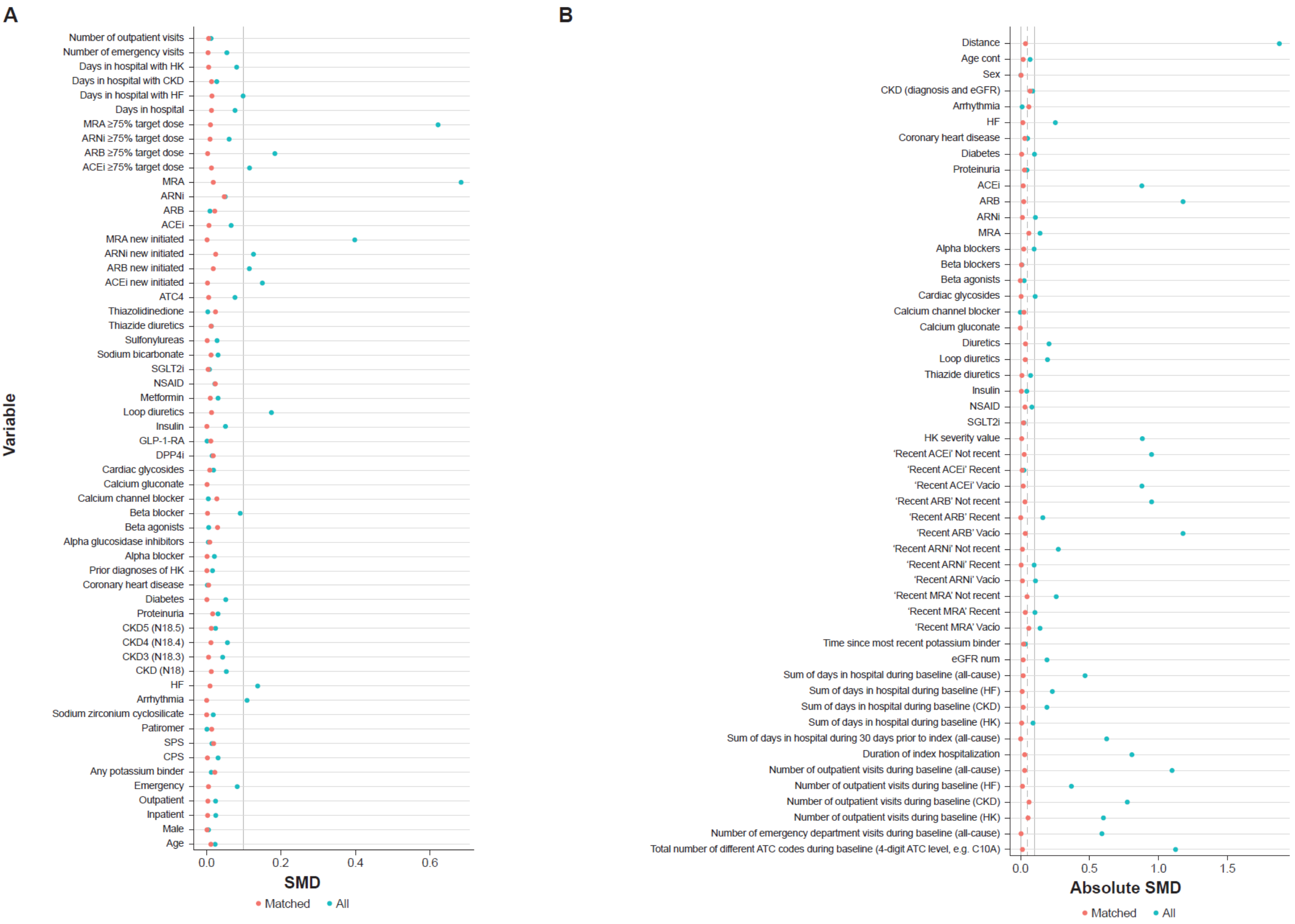

C

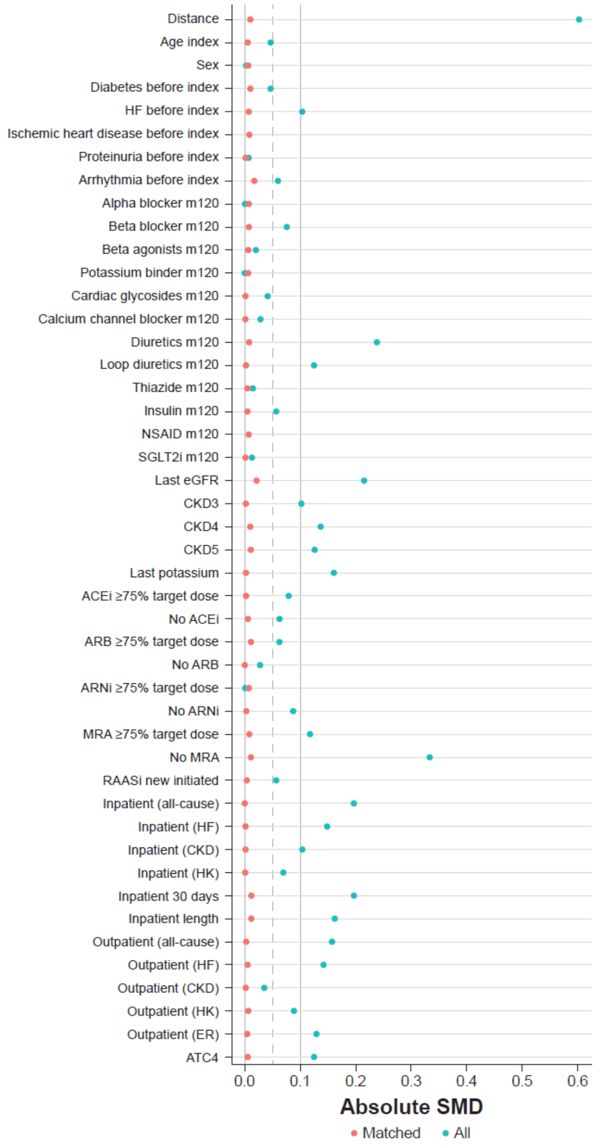

D

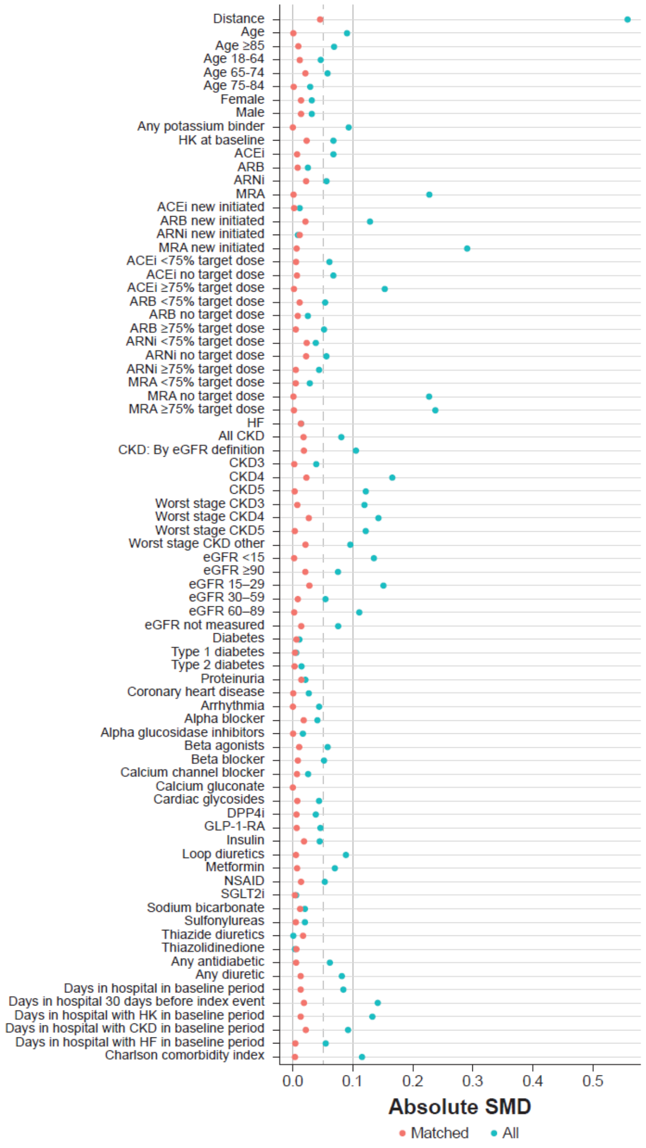

ACEi, angiotensin-converting enzyme inhibitor; ARB, angiotensin receptor blocker; ARNi, angiotensin receptor-neprilysin inhibitor; ATC, Anatomical Therapeutic Chemical; CKD, chronic kidney disease; CPS, calcium polystyrene sulfonate; DPP4i, dipeptidyl peptidase-4 inhibitor; eGFR, estimated glomerular filtration rate; ER, emergency room; HF, heart failure; HK, hyperkalemia; GLP-1-RA, glucagon-like peptide-1 receptor agonist; MRA, mineralocorticoid receptor antagonist; NSAID; non-steroidal anti-inflammatory drug; SGLT2i, sodium-glucose transport protein 2 inhibitor; SMD, standardized mean difference; SPS, sodium polystyrene sulfonate.

Supplemental Figure 3. Distribution of propensity scores before and after matching in (A) Germany, (B) Spain, (C) Sweden, and (D) the UK

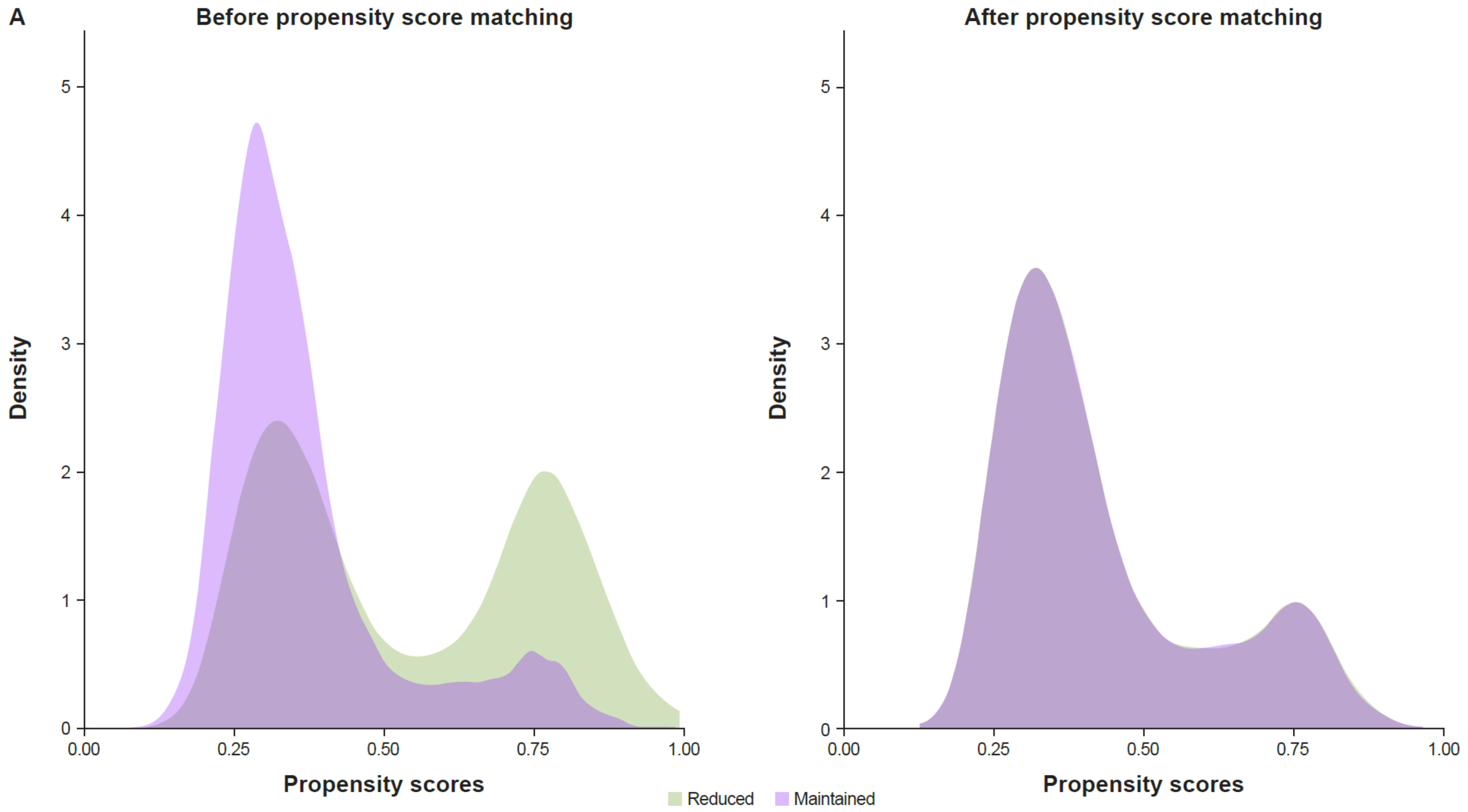

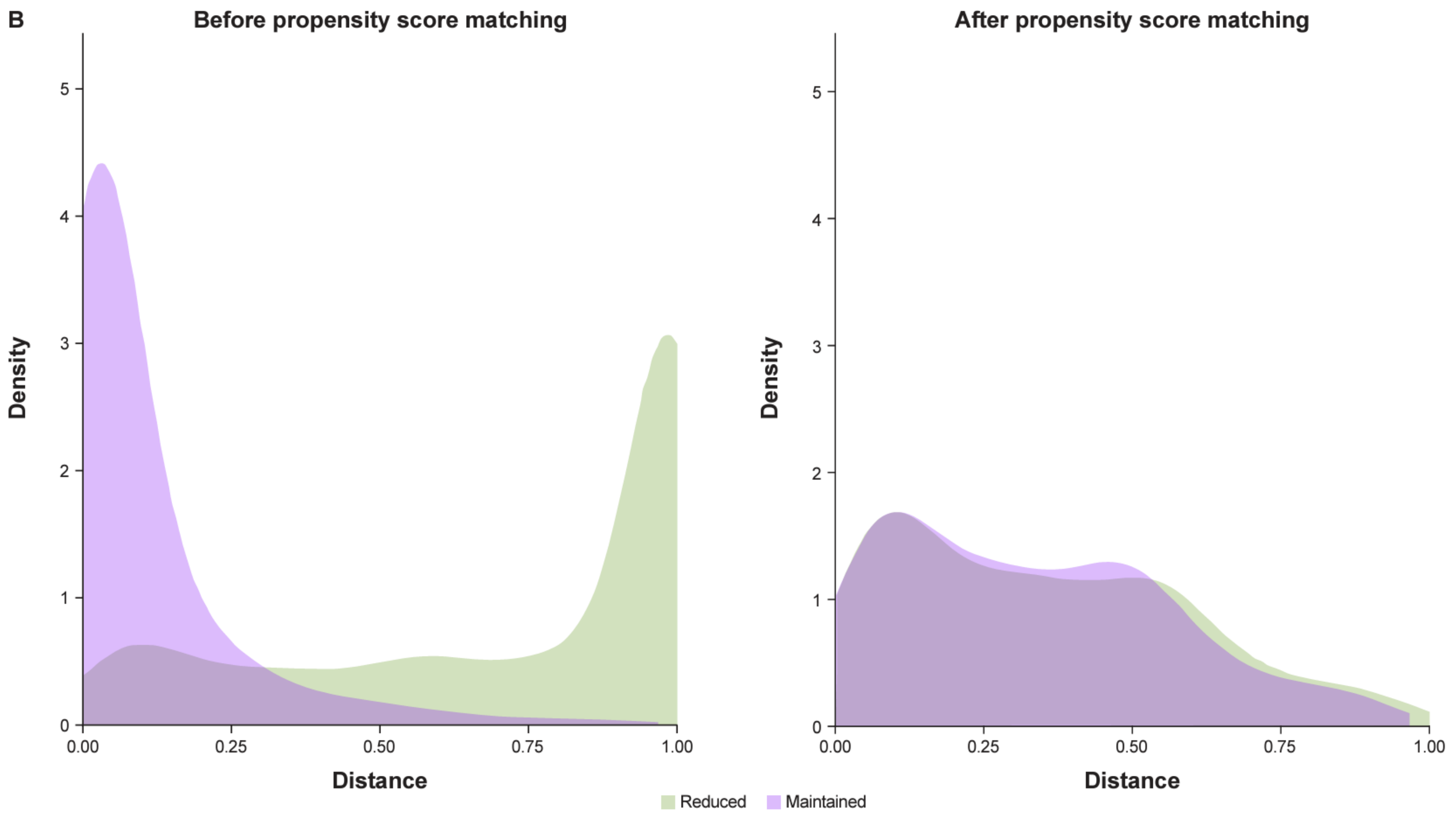
B
Before propensity score matching
After propensity score matching
Density
Distance
0
1
2
3
4
5
0.00
0.25
0.50
0.75
1.00
Reduced
Maintained

C

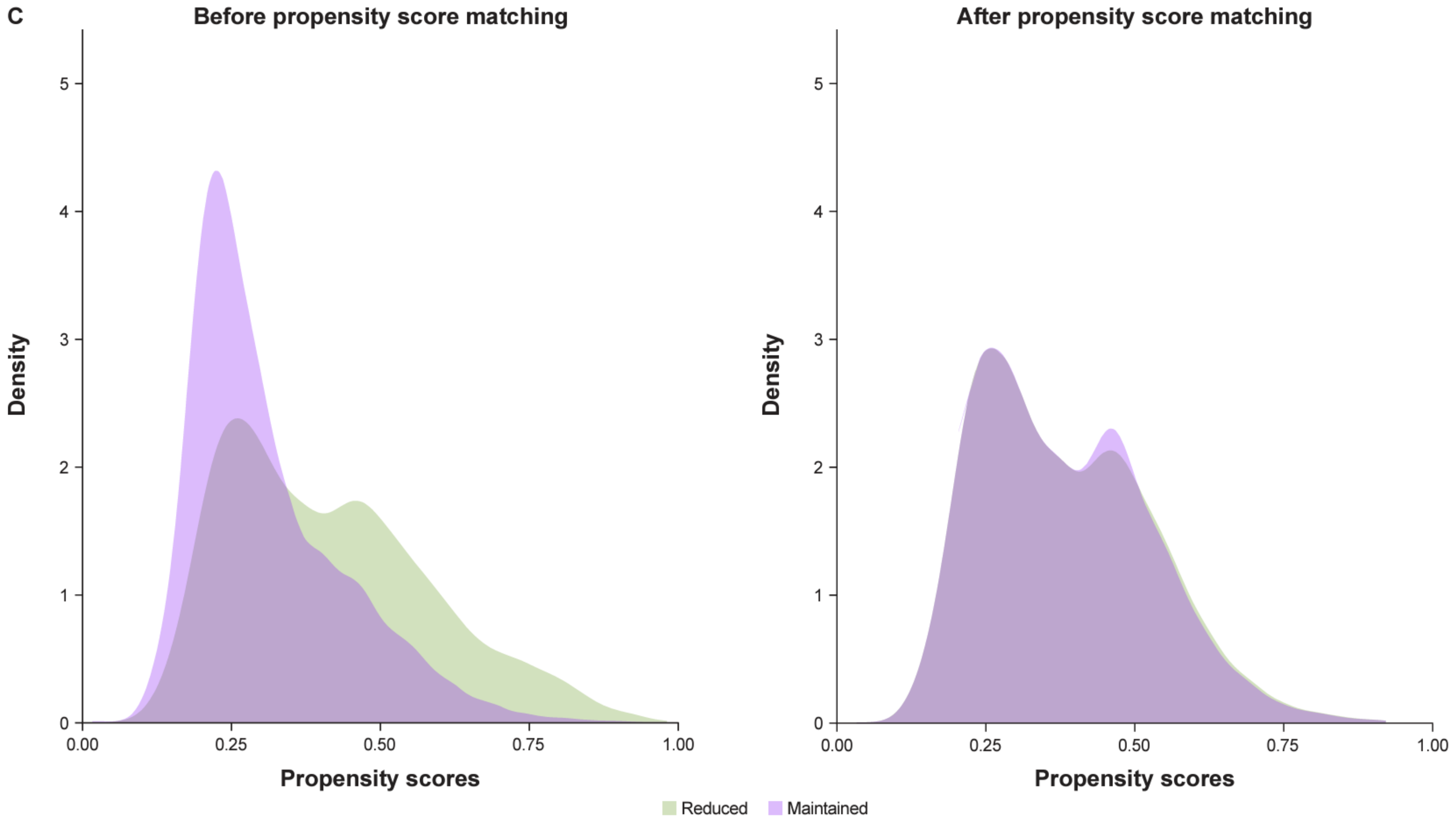

Before propensity score matching
Density
5
4
3
2
1
0
0.00
0.25
0.50
0.75
1.00
Propensity scores
After propensity score matching
Density
5
4
3
2
1
0
0.00
0.25
0.50
0.75
1.00
Propensity scores
Reduced
Maintained

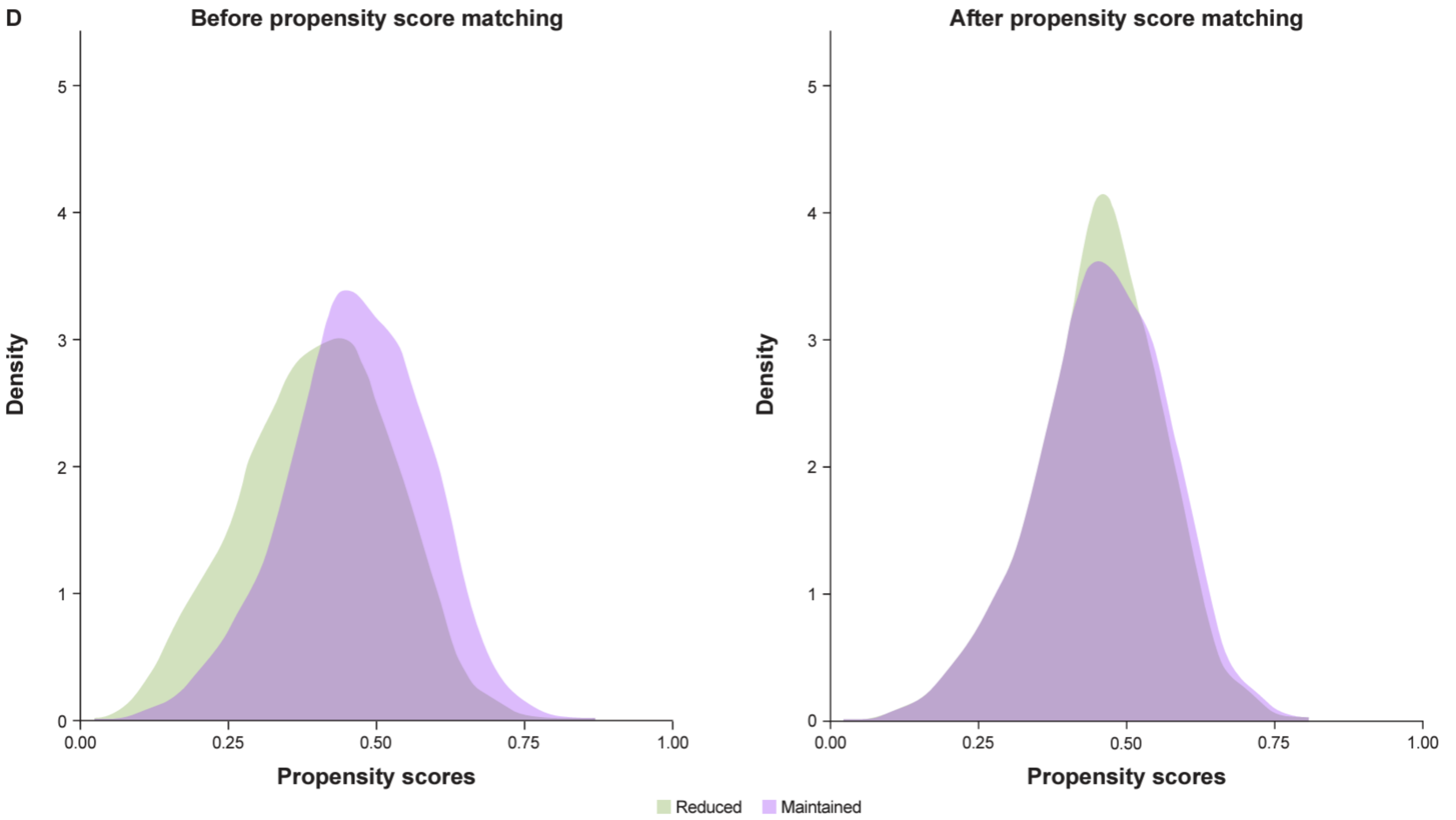

D
Before propensity score matching
After propensity score matching
Density
0
1
2
3
4
5
0.00
0.25
0.50
0.75
1.00
Propensity scores
Reduced
Maintained

References

1. WIG2: WIG2 - Scientific Institute for Health Economics and Health System Research, Available at: https://www.wig2.de/. Accessed December 21, 2023

2. Sicras-Mainar A, Sicras-Navarro A, Palacios B, Varela L, Delgado JF. Epidemiology and treatment of heart failure in Spain: the HF-PATHWAYS study. *Rev Esp Cardiol (Engl Ed)*. 2022;75(1):31–38. doi:10.1016/j.rec.2020.09.033.

3. Wolf A, Dedman D, Campbell J, Booth H, Lunn D, Chapman J, Myles P. Data resource profile: Clinical Practice Research Datalink (CPRD) Aurum. *Int J Epidemiol*. 2019;48(6):1740–1740g. doi:10.1093/ije/dyz034.
